# Insight into Hypoxia Tolerance in Cowpea Bruchid: Metabolic Repression and Heat Shock Protein Regulation via Hypoxia-Inducible Factor 1

**DOI:** 10.1371/journal.pone.0057267

**Published:** 2013-04-12

**Authors:** Ji-Eun Ahn, Xin Zhou, Scot E. Dowd, Robert S. Chapkin, Keyan Zhu-Salzman

**Affiliations:** 1 School of Agriculture and Biology, Shanghai Jiaotong University, Shanghai, China; 2 Department of Entomology, Texas A&M University, College Station, Texas, United States of America; 3 Institute of Plant Genomics and Biotechnology, Texas A&M University, College Station, Texas, United States of America; 4 MR DNA Molecular Research, Shallowater, Texas, United States of America; 5 Program in Integrative Nutrition and Complex Diseases, Texas A&M University, College Station, Texas, United States of America; U. Kentucky, United States of America

## Abstract

Oxygen is of fundamental importance for most living organisms including insects. Hermetic storage uses airtight containment facilities to withhold oxygen required for development, thus preventing damage by insect pests in stored grain. Cowpea bruchid (*Callosobruchus maculatus*) ceases feeding and growth when exposed to 2% oxygen. However, although population expansion is temporarily arrested, the bruchids (especially late stage larvae) can survive extended periods of hypoxia and recover development if normoxic conditions resume, an ability rarely found in mammals. To begin to understand fundamental mechanisms that enable insects to cope with oxygen deprivation, we constructed a 3′-anchored cDNA library from 4^th^ instar larvae subjected to normoxic and hypoxic treatments (respectively), and performed 454-pyrosequencing. Quality filtering and contig assembly resulted in 20,846 unique sequences. Of these, 5,335 sequences had hits in BlastX searches (E  = 10^−6^), constituting a 2,979 unigene set. Further analysis based on gene ontology terms indicated that 1,036 genes were involved in a diverse range of cellular functions. Genes encoding putative glycolytic and TCA cycle enzymes as well as components of respiratory chain complexes were selected and assessed for transcript responses to low oxygen. The majority of these genes were down-regulated, suggesting that hypoxia repressed metabolic activity. However, a group of genes encoding heat shock proteins (HSPs) was induced. Promoter analyses of representative *HSP* genes suggested the involvement of hypoxia-inducible transcription factor 1 (HIF1) in regulating these hypoxia-induced genes. Its activator function has been confirmed by transient co-transfection into S2 cells of constructs of HIF1 subunits and the *HSP* promoter-driven reporter.

## Introduction

In grain production, bringing in the harvest does not end the possibility of losses caused by herbivorous insects. Successful management of stored grain insects is the final component of the struggle to limit insect losses in agricultural production [1]. Like most aerobic organisms, terrestrial insects live under an atmospheric environment containing 20–21% oxygen, 79% nitrogen, and a trace of carbon dioxide and other gases. They need oxygen to survive because it is essential for generation of catabolic ATP. Availability of oxygen is therefore critical for insect growth and development. Hermetic storage is a widely adopted control measure that preserves grains by limiting availability of oxygen for insect development. In hermetic storage, dry food crops are stored in airtight facilities, so that a low oxygen (hypoxia) and/or high carbon dioxide (hypercapnia) atmosphere is obtained [2], [3]. This general method can be grouped into two subtypes: organic hermetic storage and gas-hermetic storage. The former relies on the natural respiration process of insects in dry commodities to reduce oxygen to a level which cannot sustain their lives, while the carbon dioxide level rises very substantially. In gas-hermetic storage, carbon dioxide or nitrogen is introduced into the container to drive out the air and its oxygen. Such a hypoxic environment is intended to cause insects to cease feeding, growing and reproducing, and eventually to die.

Hypoxia tolerance however, has been observed in storage insect pests. Insects that develop in stored products, a confined environment where hypoxic conditions can be created, often are able to adapt to the suboptimal conditions and to survive long periods of oxygen deprivation during their life cycles. The red flour beetle (*Tribolium castaneum*) is capable of developing higher tolerance to hypoxia under laboratory selection [4]. The hypoxia-resistant strains exhibit different characteristics than the original strain from which they developed [5]. They are heavier and have an extended life cycle and lower respiratory rate. The effect of hypoxia on another agricultural storage pest, *Tenebrio molitor* was also studied [6], [7]. Hypoxic *Tenebrio* exhibits a slower growth rate, higher mortality, more molts and a higher percentage of females than in normoxia.

The cowpea bruchid (*Callosobruchus maculatus*) is the principal pest of cowpea and some other legume seeds [8]. Its life cycle consists of egg, four larval instars, pupa and adult stages. The insect spends its entire feeding stage (i.e. 1^st^ to 4^th^ instar larva) inside the seed, consuming and damaging the whole, sound grain. Its high reproductive capacity and short life cycle can lead to a total loss of stored grain within a few months. Our previous studies showed that insects cease feeding and growth when treated with 2% oxygen +18% carbon dioxide +80% nitrogen [9]. However, they are able to cope with such stress and recover normal development once returned to a normoxic environment. This ability was particularly strong in the late larval stage, even though the actively-feeding larval stage presumably has the highest oxygen demand, and thus ought to be most susceptible to oxygen deprivation [9]. Consistently, Mbata et al. showed that sensitivity of cowpea bruchids to low oxygen created by low pressure varied with life stages [10]. Only the adult population is effectively controlled by this method, not the immature life stages [10]. Perhaps during their within-seed development, the bruchid larvae encounter hypoxic and hypercapnic conditions caused by their own respiration and respiration of the grain, and may have evolved an adaptive strategy to handle hypoxia that is associated with their specific habitat. Tolerance to low oxygen poses a difficulty in using hermetic storage as an environmentally-friendly means to control insect pests.

The molecular mechanisms of hypoxia tolerance in storage insect pests are unknown. Studies on the model insect *Drosophila melanogaster* have shed some light on how invertebrate species withstand and recover from oxygen limitation. The hypoxia-inducible transcription factor 1 (HIF1) is a critical regulator of cellular and systemic response to low oxygen [11]–[15]. HIF1 consists of two subunits α and β, and is a member of the basic helix-loop-helix/Per-Arnt-Sim (bHLH/PAS) family of proteins. Under normoxic conditions, the HIF1α subunit is hydroxylated by the HIF oxygen sensor prolyl- and asparaginyl-hydroxylases at two conserved proline residues and an asparagine residue, leading to subsequent ubiquitination [14], [16]. Hydroxylation decreases under hypoxia, causing a decrease in HIF1α degradation. In fact, HIF1α increases within minutes of exposure of cells to hypoxia, translocates into the nucleus, heterodimerizes with the constitutively expressed HIF1β and activates HIF1-responsive genes via hypoxia-responsive element binding sites. Studies in different organisms indicate that HIF1 is a global and ubiquitous regulator in transducing reduced oxygen availability into changes in gene activity and function in a pathway conserved within many organisms, from nematodes to insects to mammals [13].

Heat shock proteins (HSPs) are among the known hypoxia-responsive genes. Although induction of *Drosophila* HSPs during hypoxia requires HIF1 [17], activation of some HSPs in *Caenorhabditis elegans* are HIF1-independent [12]. Nuclear factor-κB (NF-κB), tumor suppressor protein p53, Sp factors, and other transcription factors can mediate and modify hypoxic gene expression as well [13], [18]. Cooperation of HIF1 with these transcription factors could also link low oxygen with gene expression changes [18].

It has been proposed that hypoxia-tolerant organisms depress their metabolism in order to minimize the mismatch between ATP supply and demand. *Drosophila* represses TCA cycle and glycolytic enzymes as well as genes regulating cellular respiration during oxygen deprivation [19]. A transcription factor *hairy* was found to be responsible for the coordinated suppression of genes in TCA cycle. This is in contrast to many hypoxia-sensitive animals, where the response is to increase uptake and breakdown of glucose through anaerobic glycolysis [13]. Anaerobic glycolysis can only be a short-term attempt to restore energy homeostasis. Lack of efficient, oxidative ATP production under low oxygen and continued high ATP demand soon would result in a fatal drop in ATP concentration [13].

Using a cowpea bruchid midgut cDNA microarray platform that we previously built, we have identified 765 unique ESTs that showed two-fold or greater change after a 24 hr hypoxia/hypercapnia treatment [20]. Presumably, changes in expression of these genes help insects cope with the stress. It should be noted that, for this particular custom microarray, we only sequenced ESTs that were above the fold change cutoff, while identities of the other genes remain unknown. Many genes associated with TCA, glycolysis and cellular respiration were not among the 765 ESTs. It is possible that some of these genes are not in our EST collection, or their regulation may be below the two-fold threshold. In addition, although several HSPs were hypoxia-induced, their activation mechanism remains to be determined. In the current study, we intended to capture the bruchid genes regulated by hypoxia in tissues beyond the digestive tract, by preparing samples from entire 4^th^ instar larvae subjected to hypoxia and normoxia respectively. We constructed an unnormalized, 3′-anchored cDNA library for 454-pyrosequencing. We examined expression of metabolic genes in response to hypoxia and investigated the role of HIF1 in regulating transcript expression of representative heat shock proteins by promoter analyses.

## Materials and Methods

### Hypoxia/hypercapnia treatment

Cowpea seeds infested by cowpea bruchids at a density of five insects per seed were maintained in an environmentally controlled chamber, 25°C, 35% R.H., 20% oxygen and 0% carbon dioxide. Once the bruchids reached the mid 4^th^ instar larval stage, 20 larvae in four infested seeds (five larvae per seed) were transferred to 500 mL septum bottles (Industrial glassware, Millville, NJ). The bottles were then filled with pre-mixed gases (2% oxygen +18% carbon dioxide +80% nitrogen) and tightly sealed. The levels of oxygen and carbon dioxide were verified using a head space analyzer (Mocon – PAC CHECK® Model 325, Minneapolis, MN). A 24 hr treatment of infested seeds under this condition caused a less than 1% decrease in oxygen concentration and a less than 1% increase in carbon dioxide. Control insects were also transferred into septum bottles in the same manner, but their septum was replaced with a cotton ball. This allowed atmospheric air to diffuse, maintaining the concentrations of 20% oxygen and 0% carbon dioxide. Larvae were removed from the seeds 24 hr later and kept in RNAlater (Ambion, Austin, TX) till use.

### Construction of 3′-anchored cDNA library and 454 pyrosequencing

Poly (A)^+^ mRNA was isolated from 4^th^ instar larvae using Dynabeads Oligo(dT)_25_ according to manufacturer's instructions (Invitrogen, Carlsbad, CA). Quality of the mRNA was confirmed using an Agilent 2100 bioanalyzer RNA 6000 Pico Labchip (Agilent Technologies, Santa Clara, CA).

Dynabeads-purified mRNA (1.5 μg) from control or hypoxia/hypercapnia-treated larvae was reverse transcribed using MessageAmp II aRNA Amplifiction kit (Ambion, Austin, TX). The first strand cDNAs were reverse transcribed using biotinylated oligo(dT) B-adaptor oligonucleotide (5′- biotin-GCCTTGCCAGCCCGCTCAG(T)_17_ [A,G,C] −3′), which was followed by second strand synthesis. The double stranded cDNAs were then purified according to manufacturer's instructions.

The 3′-anchored cDNA library was constructed using the Genome Sequencer DNA Library Preparation kit (Roche Applied Science, Indianapolis, IN). Briefly, 1.4 μg of double-stranded cDNA was nebulized with 45 psi nitrogen for 1 min. The nebulized cDNA was purified using a MinElute PCR Purification kit (Qiagen, Valencia, CA) and the quality of the cDNA fragments was examined using an Agilent 2100 bioanalyzer DNA 1000 Labchip (Agilent Technologies). The cDNA fragments were blunt-ended and phosphorylated by enzymatic polishing using T4 DNA polymerase and T4 DNA polynucleotide kinase. The products were purified using a MinElute PCR Purification kit (Qiagen) followed by adaptor ligation. The multiplex A-adaptors are double-stranded oligonucleotides comprised of 454 sequencing primer A (bold), 4-base nonpalindromic sequencing key (italic) and 10-base multiplex identifier tag (underlined): control insect library top strand, 5′- **GCCTCCCTCGCGCCA** *TCAG* ACGAGTGCGT -3′ and bottom strand, 5′- ACGCACTCGT *CTGA* **TGGCGCGAGG** −3′; treatment library top strand, 5′- **GCCTCCCTCGCGCCA** *TCAG* ACGCTCGACA −3′ and bottom strand, 5′- TGTCGAGCGT *CTGA* **TGGCGCGAGG** −3′. Adaptor pairs were mixed at 5 μM each in salt buffer (100 mM NaCl, 10 mM Tris-HCl, 1 mM EDTA, pH 8.0), annealed by incubation at 65°C for 15 min and gradually cooled to room temperature. Adaptor pairs were designed in such a way to allow a directional ligation at 25°C for 15 min. The cDNA library ligated with multiplex A-adaptor was purified using a MinElute PCR Purification kit (Qiagen).

To recover the biotinylated ds cDNA fragments, purified ligation mixture was immobilized onto Library Immobilization Beads (magnetic streptavidin-coated beads, Roche Applied Science) via the biotin moiety of oligo(dT) B-adaptor and were washed twice with Library Wash Buffer. The desired single-stranded 5′-A-cDNA-B-3′ template was eluted with 125 mM NaOH, neutralized, and concentrated using a MinElute PCR Purification kit (Qiagen). 454 Pyrosequencing was performed by Research and Testing Laboratory (Lubbock, TX) based on methods previously described [21]. The raw data obtained by 454 pyrosequencing were submitted to the Sequence Read Archive (SRA) database at NCBI with accession number SRP007414.

### Data analysis

Reads obtained from the 454 GS-FLX sequencing run were filtered to remove short and low quality DNAs using the LUCY Ver. 1.19 program (http://www.tigr.org/softlab). Quality filtering was conducted using default parameters, which were at least 100 bp of good-quality sequence with at most 3% unknown bases (Ns). The high-quality reads were then assembled using the TIGR Gene Indices clustering tools (TGICL) (http://www.tigr.org/tdb/tgi/software/). The high-quality reads were first clustered based on pairwise sequence similarity, and individual clusters were then assembled to generate multiple alignments and consensus sequences (contigs) using the CAP3 assembler program [22]. Consensus sequences were obtained based on two or more ESTs that overlapped for at least 40 bp with at least 95% sequence identity and at most 30 nucleotides of mismatched overhang. ESTs not falling into any assembly were considered as singletons.

All contigs and singletons were annotated by NCBI BlastX searches using the Blast2Go Ver. 2.3.6 program (http://blast2go.de) [23]. The *E*-value cutoff was set at 1×10^−6^. Functional annotations of acceptable Blast hits were based on Gene Ontology (GO) terms associated with the Blast2Go program using Blast2Go default parameters (Blast *E*-value >10^−6^, hit sequence similarity >65%, annotation cut-off: 55, GO weight: 5).

### Quantitative RT-PCR

Selected genes from 454 sequencing were subjected to quantitative RT-PCR to evaluate the transcript responses to hypoxia/hypercapnia using the ABI Prism 7900HT Sequence Detection System (Applied Biosystems, Foster City, CA). Total RNA was extracted using a Trizol-based method (Invitrogen), from whole insects as well as from dissected midgut of the insects subjected to the same treatment as described above. RNase-free DNase I (Qiagen) treatment was performed to avoid genomic DNA contamination. Random hexamer primers were used for reverse transcription, and gene-specific primers (Primer Express, Applied Biosystems) for amplifying selected genes. Quantitative RT-PCR reactions (47 cycles of 95°C for 10 s and 59°C for 45 s following an initial incubation at 50°C for 2 min and at 95°C for 3 min) were performed in SYBR Green Master Mix (Bio-Rad). PCR amplification of an 18S rRNA gene was used for normalization of input cDNA between samples. Non-template control using untranscribed RNA confirmed that no interfering PCR products derived from genomic DNA were present. Amplification specificity was determined by dissociation curve analyses. The mean induction fold was calculated according to Chi et al. [20].

### Cloning of *CmHIF1* α and β subunits from cowpea bruchids

Total RNA extracted from cowpea bruchid 4^th^ instar larvae exposed to hypoxia/hypercapnia was reverse transcribed with degenerate yet *HIF1*-specific primers 1 and 2 (Table S1) for α and β subunits, respectively. Nested PCR was performed with two pairs of degenerate primers designed from the highly conserved bHLH and PAS domains of each subunit homologue. The primary PCR (94°C for 30 sec, 42°C for 1 min, 72°C for 1 min for 35 cycles) was conducted with primers 1 and 3 for *CmHIF1α*, and with primers 2 and 4 for *CmHIF1β*. The nested PCR (94°C for 30 sec, 42°C for 1 min, 72°C for 1 min for 35 cycles) then was performed with primers 5 and 6 for *CmHIF1α*, and primers 7 and 8 for *CmHIF1β*. All PCR fragments were subcloned into pCRII vector and DNA sequences determined.

To obtain full length cDNAs, BD SMART RACE cDNA Amplification kit (BD Biosciences Clontech, Palo Alto, CA) was used to obtain the cDNA ends. For 5′ RACE, primers 9 and 10 were used for primary and nested PCR respectively for *CmHIF1α,* and primers 11 and 12 were used for *CmHIF1β* (94°C for 30 sec, 60°C for 30 sec, 72°C for 3 min for 35 cycles). Similarly, for 3′ RACE, primers 13 and 14 for PCR and nested PCR for *CmHIF1α,* and primers 15 and 16 for *CmHIF1β* were used under the same PCR conditions. After obtaining the sequence information, the full-length coding region was obtained by RT-PCR (94°C for 30 s, 55°C for 30 s, 72°C for 3 min for 35 cycles) using primers 17 and 18 for *CmHIF1α*, and primers 19 and 20 for *CmHIF1β*. PCR fragments were then subcloned into pCRII vector, and DNA sequences were confirmed.

### Identification of transcription initiation sites of hypoxia/hypercapnia regulated *HSP* genes

To locate the transcription start site of the hypoxia/hypercapnia regulated genes, 1 μg of mRNA, isolated as above, was reverse transcribed with a modified oligo(dT) primer for 5′ RACE-PCR (94°C for 30 sec, 65°C for 30 sec, 72°C for 4 min for 35 cycles). BD SMART II A oligonucleotide, and gene-specific primers 21 and 22 were used for PCR and nested PCR amplification of *CmHSP27*, and primers 23 and 24 were used for *CmsHSP21*. All PCR fragments were subcloned into the pCRII vector and subjected to sequencing analysis.

### Cloning of the putative promoters of hypoxia/hypercapnia regulated *HSP* genes

Genomic DNA from 50 cowpea bruchid larvae were extracted as previously described [24]. A PCR-based genome walking method was performed to obtain DNA sequence upstream of the coding regions of *CmHSP27* and *CmsHSP21* (Universal GenomeWalker kit; BD Biosciences Clontech). The nested PCR reactions (7 cycles of 94°C for 25 sec/70°C for 3 min, followed by 35 cycles of 94°C for 25 sec/65°C for 3 min) were performed with the adapter primers (AP1 and AP2) and gene-specific primers 21 and 22 for *CmHSP27*, or 25 and 26 for *CmsHSP21*. All PCR products were then ligated to pCRII vector and subjected to DNA sequencing analysis.

To locate potential *cis*-regulatory elements in the putative promoters of hypoxia/hypercapnia regulated genes, computer analyses of the genomic DNAs were performed using MATInspector [25] at http://www.genomatix.de/en/index.html.

### Construction of LUC reporter and expression plasmids

The 5′ flanking regions of transcription initiation sites of hypoxia/hypercapnia-regulated genes were PCR amplified (94°C for 30 sec, 55°C for 30 sec, 72°C for 2 min for 35 cycles). Oligonucleotide primer sets 27 and 28 were specific for *CmHSP27*, and 29 and 30 for *CmsHSP21*. The PCR products, restricted with *Sac* I or *Mlu* I, and *Hind* III (underlined) were subcloned into pGL3-Basic vector (Promega, Madison, WI), a vector that harbors the firefly luciferase (LUC) reporter gene. The resulting constructs, pGL3-CmHSP27/LUC and pGL3-CmsHSP21/LUC were sequenced.

To make the *pAc5-CmHIF1α* and *pAc5-CmHIF1β* constructs, the entire coding regions were amplified by PCR (94°C for 30 sec, 60°C for 30 sec, 72°C for 3 min for 35 cycles) with primers 31 and 32 for *CmHIF1α* and primers 33 and 34 for *CmHIF1β. Not* I, *Xba* I, *Kpn* I and *Xho* I (underlined) restriction sites were introduced into primers for directional cloning. After restriction digestion, the PCR fragment was ligated to pAc5.1-V5/HisA expression vector (Invitrogen), and correct DNA sequence was verified.

Deletion constructs lacking a 17 or 18 bp *HRE* site were initiated by PCR reactions using pGL3-CmsHSP21/LUC as templates, respectively primed by oligonucleotides shown in Table S1. The underlined nucleotides indicate the *HRE* deletion site. Two separate primary PCR reactions (94°C for 30 sec, 55°C for 30 sec, 72°C for 1 min 30 sec for 35 cycles) were performed with primer sets specific for each *HRE*-deletion (Table S1). Equal amounts of purified PCR products were mixed and subjected to a secondary PCR (94°C for 30 sec, 55°C for 30 sec, 72°C for 2 min for 35 cycles) with primer sets 29 and 30 for *CmsHSP21ΔHRE1* and *CmsHSP21ΔHRE2*. Sequential removal of the two *HRE* sites resulted in *CmsHSP21ΔHRE1&2*. The secondary PCR fragments were then subcloned into *Mlu* I and *Hind* III sites of pGL3-Basic. To confirm deletions of *HRE* sites, the resulting constructs, pGL3-CmsHSP21ΔHRE1/LUC, pGL3-CmsHSP21ΔHRE2/LUC, and pGL3-CmsHSP21ΔHRE1&2/LUC were sequenced.

### Transient transfection and LUC assays

Transfection experiments were performed as previously described [24]. *Drosophila* Schneider 2 (S2) cells were maintained in Shields and Sang M3 insect medium (Sigma, St. Louis, MO) with supplements at 27°C. Cells were seeded at a density of 1×10^6^ cells per well on a six-well titer plate at least one hr prior to transfection. Then a mixture of 8 μg of a reporter plasmid, 0.5 μg of each expression plasmid, and 0.2 μg of pRL-SV40 (Promega) was introduced to the cells by the calcium phosphate DNA precipitation method. pRL-SV40 reporter vector containing a cDNA encoding *Renilla* luciferase functioned as the internal control to normalize transfection efficiency. Appropriate amounts of pAc5.1/V5-HisA vector alone were added to transfection reactions to ensure comparable total DNA amounts in transfected S2 cells. Transfection assays were repeated three times or more.

After 24 h, LUC assays were performed using the Dual-Luciferase Reporter 1000 Assay System (Promega). Harvested cells were lysed in 250 μL of 1x passive lysis buffer by three cycles of freezing-thawing. After centrifugation at 15,000 g for 1 min, cell extract (20 μL) was mixed with LAR II substrate/buffer (100 μL) in a 96-well plate. Firefly LUC activity was measured using a luminometer (Victor X3 Multilabel Plate Reader, PerkinElmer, Shelton, CT). This was then followed by addition of Stop & Glo reagent (100 μL) to measure *Renilla* LUC activity. Nontransfected cell extracts were also tested to determine background signal for both firefly and *Renilla* LUC. Ratio of firefly LUC reading and *Renilla* LUC reading was calculated as LUC activity. A paired samples *t* test was used to evaluate the LUC activity data using SPSS for Windows 12.0.1.

## Results and Discussion

### Pyrosequencing, data assembly, homology search and gene ontology

To capture genes altered under hypoxic/hypercapnic conditions, we used insects exposed to low oxygen and high carbon dioxide as well as normoxic insects as samples for mRNA extraction to establish a transcriptome. Sequencing 3′ cDNA fragments generated by nebulization is reported to be able to overcome the biased representation derived from 454 pyrosequencing [26]. We used biotinylated oligo(dT) oligonucleotide for cDNA synthesis, and for recovery of the double stranded cDNA fragments after nebulization via magnetic streptavidin-coated beads. The library thus only comprises the 3′-end cDNAs fragments with the size mostly concentrated at 500 bp, as indicated by RNA HighSense Chip analysis (Bio-Rad, Hercules, CA). Such even lengths of 3′ cDNAs can avoid bias due to incomplete reverse transcription of the mRNAs, and require no adjustment for transcript length [26]. Based on our previous experience related to large scale sequencing of our cowpea bruchid EST collection, we believe that for most genes, a 500 bp fragment upstream of poly(A) tail should cover the 3′-UTR as well as some coding regions. We expected that although transcriptome sequencing information is lacking for this species, sufficient coding sequences would be generated by 454 sequencing to allow for gene identification through sequence database searches.

**Figure 1:**
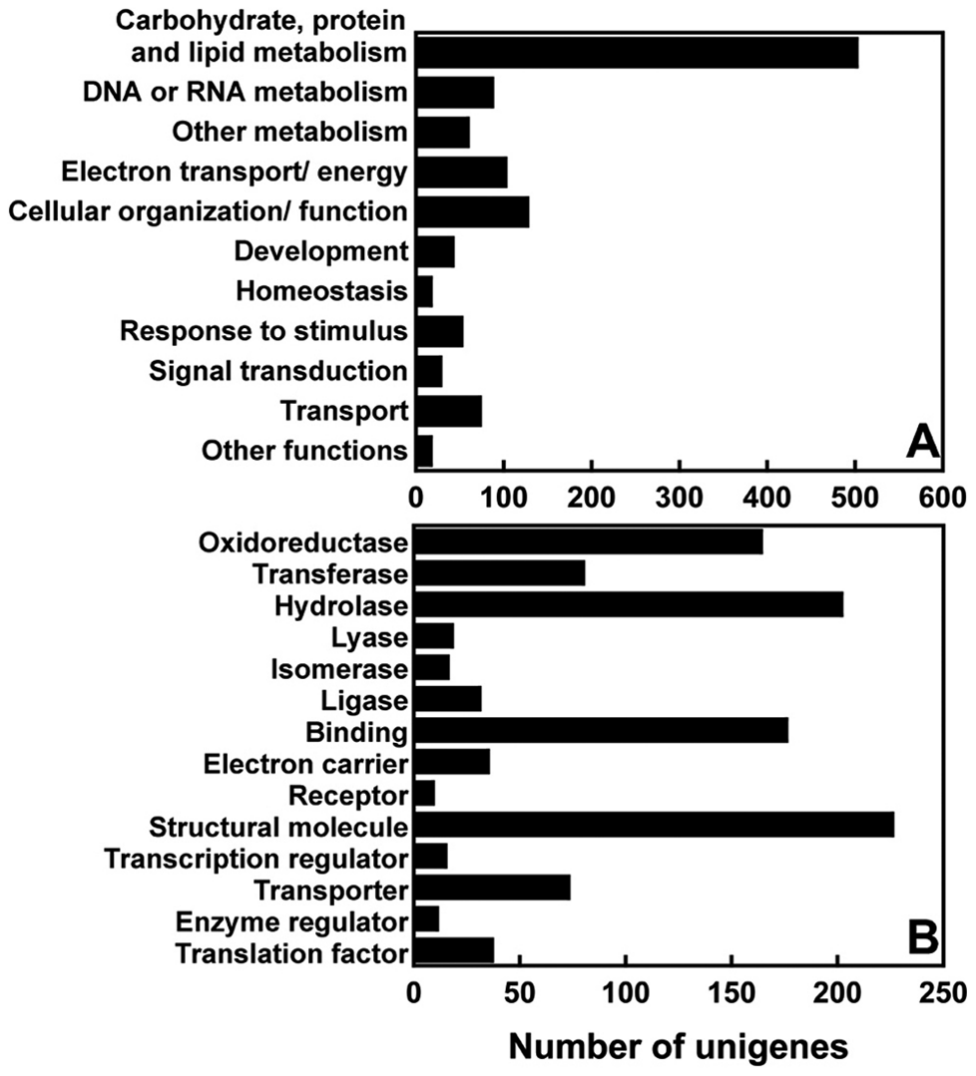
Functional distribution of the 1,036 GO annotated ESTs derived from assembly and homology search of 454-sequencing reads. Shown are GO terms of (A) biological process and (B) molecular function.

Sequencing of the 3′-anchored cDNAs from normoxic and hypoxic insects via 454 GS-FLX runs resulted in a total of 275,358 reads (Table 1). After filtering to eliminate short and low quality sequences using the LUCY program [27], we obtained 217,827 high-quality reads with average DNA lengths of 225 bp. Assembly using the TIGR Gene Indices clustering tools [28] formed a total of 13,340 contigs (average size 347 bp), leaving 7,506 unassembled singletons (average size 224 bp). The 20,846 contig and singleton sequences were submitted for similarity search by NCBI BlastX using the Blast2Go program. Of these, 5,335 ESTs had hits exceeding our threshold (*E*-value <10^−6^). As a result, 2,979 unigenes were identified. Gene ontology (GO) was performed to classify the 2,979 unigenes based on biological process and molecular function (Fig. 1). A total of 1,036 unigenes were annotated for GO terms. The most abundantly expressed genes were involved in protein, carbohydrate and lipid metabolism (502 genes). Genes involved in cellular organization/function (127 genes), electron transport/energy pathway (102 genes) and response to stimulus (52 genes) were also abundantly expressed.

**Table 1:** Summary of 454-sequencing, assembly, BLAST search and gene ontology analysis.

454 Sequencing	
Total 454 reads	275,358
Total quality reads after quality-filtering	217,827
Assembly	
Total contigs (average size)	13,340 (347 bp)
Total singletons (average size)	7,506 (224 bp)
Similarity search & annotation	
Total contigs & singletons with BLASTX hits (*E*-value <10^−6^)	5,335
Total unigenes expressed	2,979
Total unigenes with GO annotations	1,036

The remaining 15,511 ESTs that did not return any BlastX hits most likely can be grouped into three categories: (1) The fragment is part of the long 3′-UTR. Due to its gene- and/or isoform-specific nature, no homology was found; (2) The fragment contains open reading frame from a known gene, but is from a highly variable coding region; (3) The fragment encodes a new uncharacterized protein. For non-model systems which lack genomic sequences, the sequencing of 3′ cDNAs, although cost-effective, has limits in EST characterization. Sequencing entire cDNAs, rather than focusing on the 3′ end, will improve gene identification in categories (1) and (2). Nevertheless, our 454-sequencing results have provided a reservoir from which genes of interest can be selected for further investigation.

### General down-regulation of metabolic genes

When oxygen is not limited, oxidative phosphorylation is the principal ATP supply for eukaryotic cells. Under hypoxic challenges, many hypoxia-sensitive animals or mammalian organs like brain, kidney and heart, activate multiple genes that function to restore energy and oxygen homeostasis. Such an adaptation utilizes induced genes in the glycolytic pathway for energy production and stimulates angiogenesis and erythropoiesis to increase tissue oxygenation. Such an approach to compensation for ATP demand can only be short-term. Anaerobic glycolysis increases toxic end products while still failing to meet the cell's need for ATP [29], [30]. In hypoxia-tolerant organisms or stages of development, survival strategies are based on energy conservation rather than on energy compensation. Reduced and balanced ATP supply and consumption prevents lethal falls in cellular ATP levels [13].

**Figure 2:**
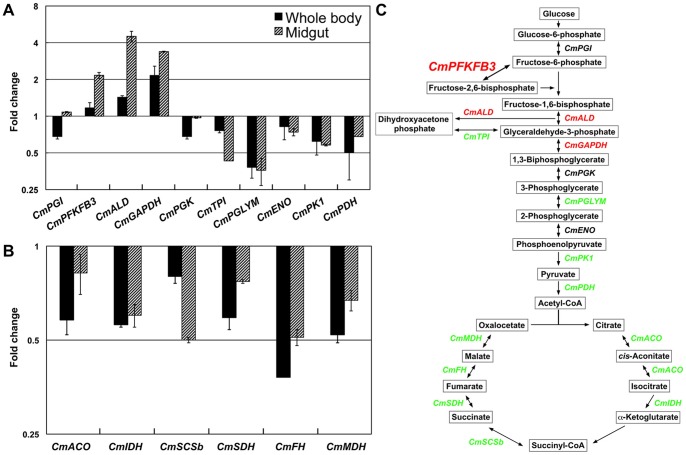
Gene expression profiles of (A) glycolytic and (B) TCA cycle enzymes in response to hypoxia/hypercapnia in cowpea bruchid larvae. Total RNA was extracted from whole insect body as well as from midgut of cowpea bruchids exposed to hypoxia/hypercapnia for 24 hr. qRT-PCR was performed as described in Materials and Methods. (C) Schematic illustration of the gene expression changes based on qRT-PCR. Green: down-regulated; red: up-regulated; black: no change (below the cutoff of 1.5 for both tissues). *CmPGI*: phosphoglucose isomerase, *CmPFKFB3*: 6-phosphofructo-2-kinase/fructose-2,6-bisphosphatase, *CmALD*: fructose 1,6-bisphosphate aldolase, *CmGAPDH*: glyceraldehyde-3-phosphate dehydrogenase, *CmPGK*: phosphoglycerate kinase, *CmTPI*: triosephosphate isomerase, *CmPGLYM*: phosphoglycerate mutase, *CmENO*: enolase, *CmPK1*: pyruvate kinase isoform 1, *CmPDH*: pyruvate dehydrogenase, *CmACO*: aconitase, mitochondrial, *CmIDH*: isocitrate dehydrogenase, *CmSCSb*: succinyl-CoA synthetase beta chain, *CmSDH*: succinate dehydrogenase, *CmFH*: fumarate hydratase, mitochondrial precursor, *CmMDH*: malate dehydrogenase.

**Figure 3:**
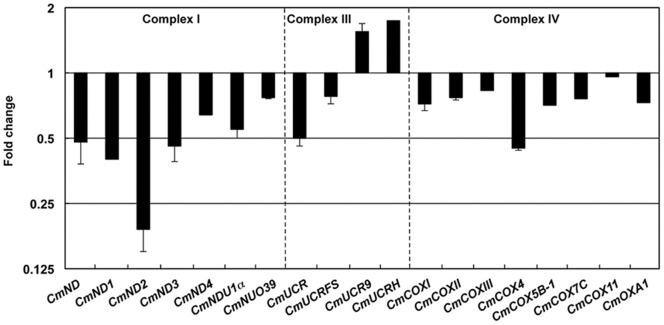
General suppression of genes encoding components of mitochondrial electron transport chain by hypoxia/hypercapnia in cowpea bruchid larvae. qRT-PCR was performed to profile gene expression in each complex of the electron transport chain after cowpea bruchids were exposed to hypoxia/hypercapnia for 24 hr. *CmND*: NADH dehydrogenase, *CmND1*: NADH dehydrogenase subunit 1, *CmND2*: NADH dehydrogenase subunit 2, *CmND3*: NADH dehydrogenase subunit 3, *CmND4*: NADH dehydrogenase subunit 4, *CmNDU1α*: mitochondrial NADH dehydrogenase (ubiquinone) 1 alpha subcomplex, *CmNUO39*: NADH-ubiquinone oxidoreductase 39 kda subunit, *CmUCR*: ubiquinol-cytochrome c reductase complex, CmUCRFS: ubiquinol-cytochrome c reductase iron-sulfur subunit, *CmUCR9*: ubiquinol-cytochrome c reductase subunit 9, *CmUCRH*: mitochondrial ubiquinol-cytochrome c reductase hinge protein, *CmCOXI*: cytochrome c oxidase subunit I, *CmCOXII*: cytochrome c oxidase subunit II, *CmCOXIII*: cytochrome c oxidase subunit III, *CmCOX4*: mitochondrial cytochrome c oxidase subunit 4, *CmCOX5B-1*: mitochondrial cytochrome c oxidase subunit 5B isoform 1, *CmCOX7C*: mitochondrial cytochrome c oxidase subunit 7C, *CmCOX11*: cytochrome c oxidase assembly protein cox11, *CmOXA1*: cytochrome c oxidase biogenesis protein OXA1.

To determine how hypoxia impacts ATP production in cowpea bruchids, we assessed transcript levels of 10 genes encoding glycolysis-related enzymes and six genes encoding TCA cycle enzymes by qPCR under normoxia and hypoxia. A majority of these genes were down-regulated or showed no changes (Fig. 2). Also examined were 19 genes involving cellular respiration (Fig. 3). Again, more genes encoding components of the mitochondrial electron transport chain were down-regulated than up-regulated, suggesting that reduced and balanced ATP production and demand is an important strategy for hypoxia tolerance in cowpea bruchids as well.

It should be noted that we failed to recognize the majority of these tested genes in our previous microarray study, most likely due to their lower than the two-fold expression change, therefore these were not sequenced previously even if they were among our cDNA collection. Our current 454-results, however, made these sequences readily available for gene expression studies.

Interestingly, although expression patterns of the above metabolic genes are largely in agreement with what was observed in *Drosophila*, differences exist. In *Drosophila*, genes encoding glycolytic enzymes are repressed [19], while in cowpea bruchids, particularly in the midgut, microarray and qPCR analyses identified phosphofructo-2-kinase/fructose-2,6-bisphosphatase 3 (*CmPFKFB3*) as a hypoxia-induced gene (Fig. 2). In addition, glyceraldehyde-3-phosphate dehydrogenase (*CmGAPDH*) and fructose 1,6-bisphosphate aldolase (*CmALD*) were induced in cowpea bruchids. The bifunctional enzyme PFKFB3 is known to be a key regulator of glucose flux through the glycolytic pathway [31]. This particular isoform has the highest kinase:phosphatase activity ratio [32]. The resulting elevated fructose 2,6-bisphosphate levels in turn promote high glycolytic rates in the cell. Differential expression of these glycolytic enzyme genes raises the question of whether cowpea bruchids are able to adapt to environmental hypoxia partly by switching from aerobic respiration to anaerobic glycolysis. Despite the fact that *Drosophila* exhibits remarkable resilience to oxygen deprivation, this model species normally does not live in extremely low oxygen atmospheres. The adaptive strategies may differ, at least partly, in species that do need to frequently deal with low oxygen stress. Further studies are needed to elucidate the significance of the up-regulation of the glycolysis-related genes.

### Cowpea bruchid HIF1 (CmHIF1) subunits share high sequence similarity and domain structure with homologues

**Figure 4:**
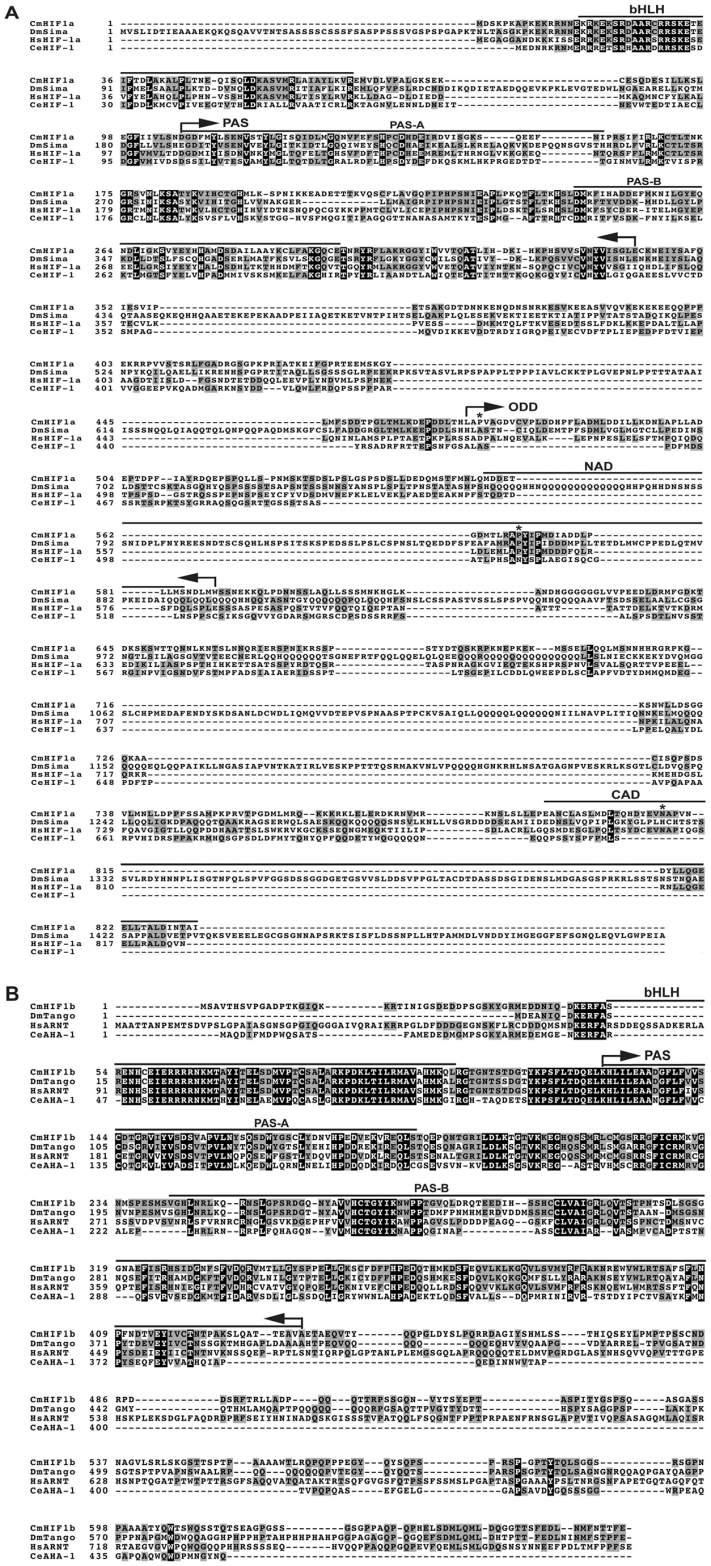
CmHIF1 subunits share high sequence similarity with homologues. Shown are protein sequences deduced from full length cDNAs of (A) *CmHIF1α* ) (GenBank accession number JN228344) and (B) *CmHIF1β* (GenBank accession number JN228345). For both *α* and *β* subunits, the bHLH, domains, and PAS-A and PAS-B regions are marked with lines above sequences. The boundaries of the PAS domains are indicated by bent arrows. The CmHIF1α-specific ODD (oxygen-dependent degradation) domain, NAD (N-terminal transactivation domain) and CAD (C-terminal transactivation domain) are also indicated by bent arrows and lines above the sequences, respectively. Prolyl and asparaginyl residues predicted to be hydroxylated by prolyl- and asparaginyl-hydroxylases are marked by asterisks. DmSima (GenBank accession number U43090), HsHIF-1α (U22431) and CeHIF-1 (NM_001028270) are HIF-1α members from *D. melanogaster*, *H. sapiens* and *C. elegans*. Similarly, DmTango (AF020426), HsARNT (M69238) and CeAHA-1 (AF039569) are respective HIF-1β members from the above species. Amino acid residues identical across all sequences are shaded black, and identical across some sequences are shaded grey.

Although our 454-sequence collection made possible the study of metabolic gene expression in response to hypoxia, it did not reveal any apparent *CmHIF1* fragments. Given the important role of this transcription factor in regulating cellular and systemic response to low oxygen, we cloned putative α and β subunits using nested and RACE PCR techniques, followed by further investigation of its regulatory function. These subunits share high sequence similarity and domain structure with homologues from *Drosophila*, *C. elegans* and human. *CmHIF1α* (2,890 bp) contains an open reading frame (ORF) that encodes a protein of 833 amino acid residues and *CmHIF1β* (4,005 bp) contains an ORF of 662 amino acid residues (Fig. 4). As expected, they contain the conserved bHLH and PAS domains. An oxygen dependent degradation domain (ODD) and the C-terminal transactivation domain (CAD) that modulate the activity of HIF1α as a function of oxygen availability [14] were also located in CmHIF1α (Fig. 4A). Like other HIF1β homologues, CmHIF1β lacked ODD and CAD domains (Fig. 4B). Presumably, it is also constitutively transcribed and translated, independently of oxygen tension [33].

### Cowpea bruchids regulate hypoxia-induced heat shock proteins via CmHIF1

**Figure 5:**
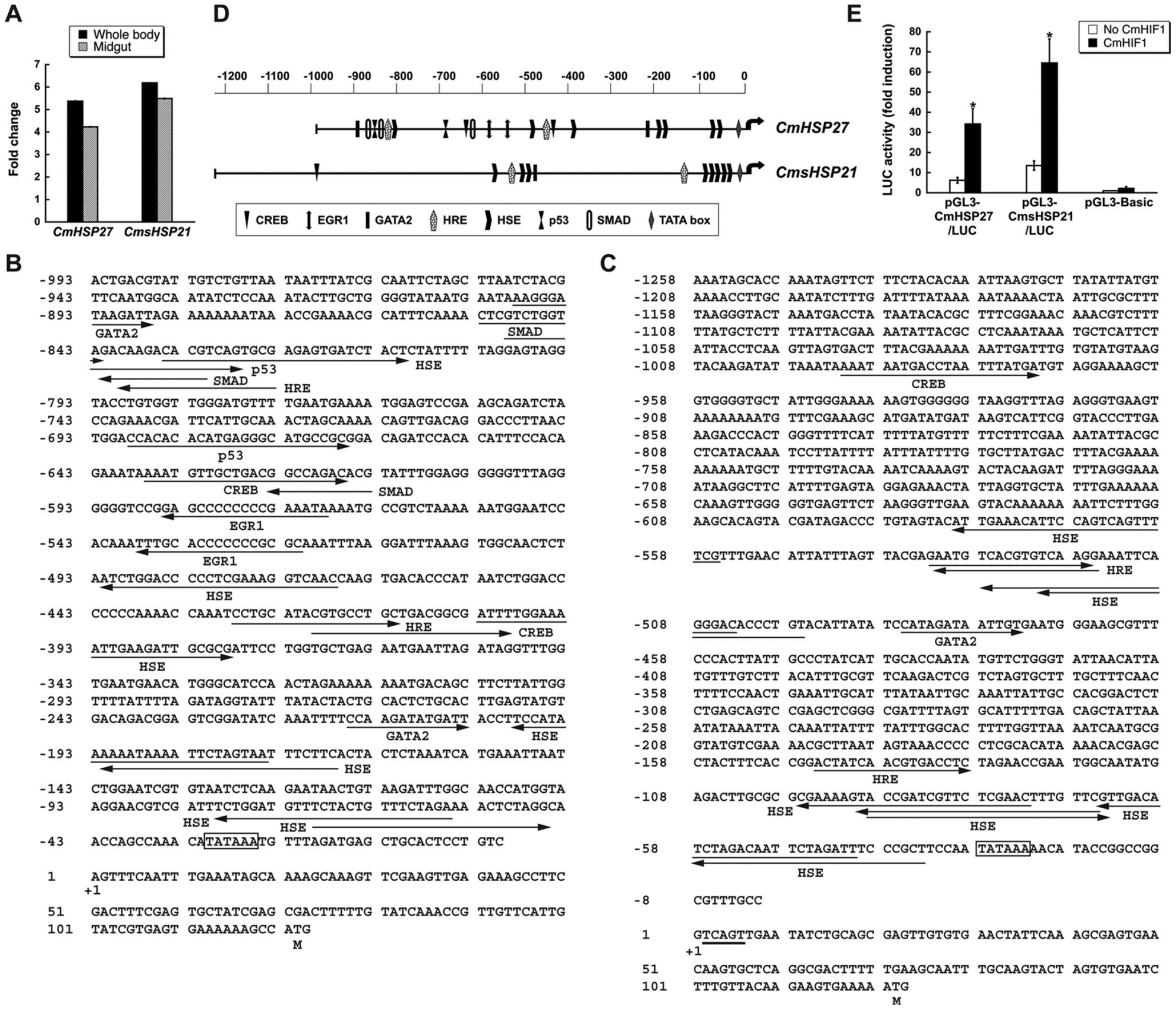
CmHIF1 enhances the promoter activity of selected *CmHSP*s. (A) Cowpea bruchids induce expression of *CmHSP27* and *CmsHSP21* in response to hypoxia/hypercapnia. After 24 hr exposure, total RNA was extracted from whole body and midgut, respectively. qRT-PCR was performed to profile gene expression of these selected heat shock proteins. (B, C) Architecture of genomic DNA upstream of *CmHSP27* (B) and *CmsHSP21* (C) coding regions. Transcription initiation site is marked as +1, and the upstream sequences are denoted with negative numbers. Potential *cis*-elements are illustrated by arrows under DNA sequence in putative *CmHSP27* and *CmsHSP21* promoters. Putative TATA boxes are boxed, and the pentamer sequence is underlined. (D) Schematic comparison of the promoter regions of *CmHSP27* and *CmsHSP21* genes. Both promoters have binding sites for HIF1, CREB, GATA2, HSF family and TATA box. (E) CmHIF1 enhances the promoter activity of *CmHSP*s in *Drosophila* S2 cells. S2 cells were cotransfected with 8 μg of LUC reporter plasmids and 0.5 μg of each expression plasmid (pAc5-CmHIF1α and pAc5-CmHIF1β; black bars) or equivalent empty expression vector (white bars), respectively. The latter was used to ensure comparable total DNA amounts in transfected S2 cells. The difference between LUC activity of reporter plasmids, pGL3-CmHSP27/LUC and pGL3-CmsHSP21/LUC showed statistical significance (*t* test, p<0.05) in the presence versus absence of CmHIF1. The reporter plasmid pGL3-Basic was included as a control for LUC activity. *Renilla* luciferase vector (pRL-SV40) was used as an internal control for transfection efficiency. Luciferase activity is expressed as fold induction relative to that of the pGL3-Basic vector. Error bars represent the means ± S.E. of four independent cotransfections.

A group of hypoxia-induced genes in cowpea bruchids are *HSP* genes [20]. Induction of *CmHSP27* and *CmsHSP21* were detected in bruchid larval midgut as well as in whole body (Fig. 5A). In *Drosophila* as well as in mammals, hypoxia induces *HSP* genes [17], [34], [35]. Upregulation of HSPs led to a significant increase in adult fly survival [35]. That HSPs function as chaperones as well as in apoptosis likely explains the increased insect survival. When comparing mRNA expression in wild-type and the *hif1* (HIF1-deficient) mutant in *C. elegans*, Shen et al. discovered HIF1-independent signaling pathways [12]. Several heat shock proteins are among the hypoxia-responsive yet HIF1-independent genes. Furthermore, a number of other transcription factors are hypoxia-responsive and able to mediate and modify hypoxic gene expression [13], [18], [36].

To determine if induction of *CmHSP* homologues involves the HIF pathway, we obtained putative promoter sequences of *CmHSP27* and *CmsHSP21* for further analysis. A 1,284 bp fragment for *CmHSP27* containing 291 bp of the coding sequence and 993 bp of upstream flanking sequence was obtained by a PCR-based genome walking method (Fig. 5B). Likewise, a 1,441 bp fragment for *CmsHSP21*, containing coding (183 bp) and 5′ flanking (1,258 bp) regions, was also cloned (Fig. 5C). The transcription initiation sites were determined by 5′ RACE-PCR. Putative TATA boxes located between −31 and −26 for *CmHSP27* and between −28 and −23 for *CmsHSP21* respectively were identified. The *CmsHSP21* gene has a TCAGT pentamer, known as the arthropod initiator sequence that is important for promoter functions [37], [38].

Searching for potential *cis*-elements revealed hypoxia-responsive elements (*HRE*s), possible binding sites for HIF1 (Fig. 5B, 5C). Other binding sites of hypoxia-responsive transcription factors, such as CREB, EGR1, GATA2, p53 and Smad, were identified as well. *cis*-Elements common to both selected *HSP*s were *HRE, HSE, CREB*, *GATA2* elements and a *TATA* box (Fig. 5D). As expected from *HSP* promoters, there exist multiple heat shock elements (*HSE*s), the binding sites of the heat shock factor [39]. Interestingly, the transcript of heat shock factor itself, the main transcriptional regulator of stress-induced *HSPs*, was up-regulated by hypoxia through HIF1 [17].

Such motif searches suggest that HIF1 might be the transcriptional regulator of HSPs in cowpea bruchids when challenged by low oxygen. To provide conclusive evidence that the HIF pathway is involved in *CmHSP* induction by hypoxia, co-transfection into S2 cells of *HIF1* with the *HSP* promoter-reporter constructs were tested (Fig. 5E). Transient overexpression of CmHIF1 induced 5 to 6-fold increases in the luciferase (LUC) activity of pGL3-CmHSP27/LUC and pGL3-CmsHSP21/LUC. CmHIF1-mediated activation of LUC activity was not observed in the control construct (pGL3-Basic) indicating specific transactivation activity of CmHIF1. The data suggested that CmHIF1 promotes transcriptional activation of *CmHSP27* and *CmsHSP21*.

Under stressful conditions, it is not uncommon to see the upregulation of HSPs. Mouse *HSP27*, which shares high homology to our *CmHSP27* clone, is unique because it specifically responds to hypoxia in the retinal ganglion cell layer, while other heat shock proteins (like Hsp-70 and −90) were not up-regulated [40], suggesting a unique function in hypoxia in addition to general stress. The small heat shock protein sHSP family contains a group of 15–30 kDa polypeptides [41]. These can prevent protein aggregation and/or restore the biological activity of the associated proteins. Such functions are believed to mitigate protein misfolding and denaturation triggered by environmental stresses such as heat, osmotic and hypoxic injury *in vivo* [15].

**Figure 6:**
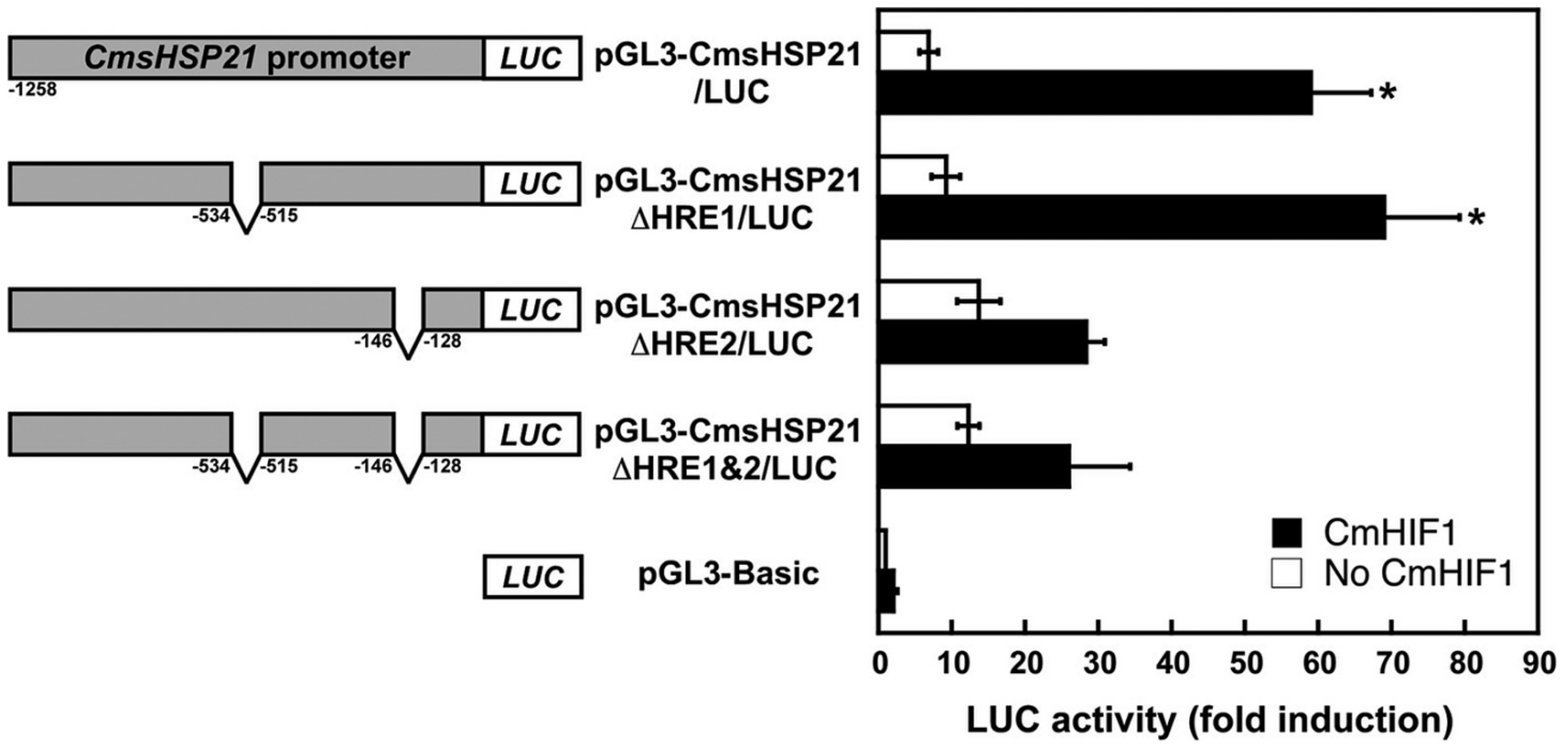
CmHIF1-mediated activation of CmsHSP21 requires binding at the *HRE2*-element. S2 cells were cotransfected with 8 μg of LUC reporter plasmids containing *HRE*-deletions and 0.5 μg of each expression plasmid (pAc5-CmHIF1α and pAc5-CmHIF1β; black bars) or equivalent empty expression vector (white bars), respectively. Numbering of schematic promoters (left panel) in reporter plasmids is relative to the transcription initiation site. Asterisks indicate significant differences in luciferase activity (right panel) of individual constructs in the presence versus absence of CmHIF1 subunits (t-test, *P*<0.05). Luciferase activity is expressed as fold induction relative to that of the pGL3-Basic vector. Error bars represent the means ± S.E. of three cotransfections. Transfection efficiency was normalized as described in Materials and Methods.

To further confirm the role of CmHIF1 in transcriptional activation of *CmsHSP*s, we selected pGL3-CmsHSP21/LUC and performed *HRE* deletion and cotransfection analyses. While deletion of the *HRE2* fragment (−145 to −129 bp) drastically reduced LUC activity, removal of *HRE1* (−533 to −516 bp) showed no impact by itself, nor did it enhance *ΔHRE2* alteration of the LUC signal (Fig. 6), suggesting that *HRE2* is a *bona fide cis*-element, essential for CmHIF1-mediated activation. Electrophoretic mobility shift assays with nuclear protein extracts from the cowpea bruchid itself will provide additional evidence for the *cis*-regulatory function of *HRE2* and for a CmHIF1 trans-activator role in our species of interest. Such an approach also has the potential to identify other previously unknown transcription factors essential for hypoxia signal transduction. It is very likely that these factors work together to coordinate expression of hypoxia-responsive genes.

## Conclusions

Oxygen is the primary source of metabolic energy for all eukaryotic cells, thus it is not surprising that insects have developed the capacity to respond to hypoxic insults. A lack of knowledge on molecular mechanisms mounted by storage pests in response to hypoxia has limited our ability to counter the adaptive responses. Next generation sequencing technology has allowed us to investigate the molecular mechanisms of insect adaptive responses. In this study, 454 sequencing provided a reservoir from which genes along the ATP-generating pathway were selected. Expression profiling of these genes further supported the notion that reduced energy supply and compensation has facilitated insects' coping with the lack of oxygen. Furthermore, induction of HIF1-mediated stress-tolerance genes also appears to be part of the survival strategy commonly shared by arthropods. As we gradually achieve a better understanding of oxygen sensing and signal transduction in storage pests, we may be able to develop control strategies to slow down resistance development in insects against the environmentally sound hermetic storage method.

## Supporting Information

Table S1 **Primers synthesized for this study.**
(DOC)
