# Supplementary material for: Insight into Hypoxia Tolerance in Cowpea Bruchid: Metabolic Repression and Heat Shock Protein Regulation via Hypoxia-Inducible Factor 1
Source: PLoS One. 2013 Apr 12;8(4):e57267. doi: 10.1371/journal.pone.0057267 (PMC3625201; doi:10.1371/journal.pone.0057267)
Supplement: Table S1 — Primers synthesized for this study. (DOC) [file pone.0057267.s001.doc]

**Table S1. Primers synthesized for this study.**

| For PCR amplification of *CmHIF1α* and *CmHIF1β* * |
| --- |
| 1. TCRATRTTBSWWGGATG (antisense) **  2. CCYTCYTTYTTBACWGT (antisense) **  3. AARGARAARTCHMGMGA (sense) **  4. AACAARATGACNGCCTAYATC (sense) **  5. GARAARTCHMGMGAYGC (sense) **  6. ATRTTBSWWGGATGMGG (antisense) **  7. ATGACNGCCTAYATCACVGA (sense) **  8. TCYTTYTTBACWGTNCC (antisense) **  9. ATAAATACATGAAATCGCCATCGTTCG (antisense)  10. GAAGCCTTCGAGTGATTTCAGGAG (antisense)  11. AGGATGGTTTGTAAGTGCCGTCGGTAC (antisense)  12. TGTTACCAGTGCCCCGAAGCTGC (antisense)  13. GGAAGATCTGTCAATCTCAAATCTGCTACT (sense)  14. GGTGATACATTGTACGGGTCACATGTT (sense)  15. CCCGTCCTCAATTACTCACAGAGCGACTGG (sense)  16. TACGGTTCCTGCCTCTACGACAAC (sense)  17. GATTGTTGTGATTGTGGGAATTATTATC (sense)  18. CGCCAGGGGCATCGACTGTTA (antisense)  19. GACCCCAACAAGGTTTTTCTAATATGG (sense)  20. ATTAAGAATTTATTGACTTTTCAAGTTTGT (antisense) |
| For 5′ RACE and 5′ flanking region PCR amplifications of *HSPs** |
| 21. CAAAATTGGCTTGGAACTTGTCCTGATTGAGCA (antisense)  22. ACCTGCTGTGTCTTGAAGGGCGGCATTTAA (antisense)  23. GCCTTCAATCTGCTTCGCTGGCTCACCCGT (antisense)  24. CTTGGCATCCAACCTAGGTGCAGTGATAGTCAG (antisense)  25. ACCTGGTAAGTAGCCTGGGGATGGTCAATGGCT (antisense)  26. CAGCAGCCTCGATGGACGATAGTGGAAAGG (antisense) |
| For LUC reporter and expression constructs* |
| 27. CAGACGAGCTCACTGACGTATTGTCTGTTAATAATTTA (sense)  28. GTATGAAGCTTGACAGGAGTGCAGCTCATCTAAAC (antisense)  29. CCGACACGCGTAAATAGCACCAAATAGTTCTTTCTACA (sense)  30. GTATTAAGCTTGGCAAACGCCGGCCGGTATGT (antisense)  31. TATAAGCGGCCGCCCACCATGGACTCTAAACCAAAGGCTCC (sense)  32. GAGCTTCTAGATATGGCGGTGTTGATGTCGAGAGCTGTGA (antisense)  33. GACCGGGTACCCCACCATGTCAGCTGTTACACATTCA (sense)  34. TCGAGCTCGAGTTCAAAGGTTGTGTTGAACATGTT (antisense) |
| For *HRE*-deletion constructs* |
| 35. GGTGTCCCTGAATTTTCGTAACTAAATAATGTTCAAACG (antisense)  36. ATTATTTAGTTACGAAAATTCAGGGACACCCTGTACATT (sense)  37. CATTCGGTTCTACCGGTGAAAGTAGGCTCGTGTTT (antisense)  38. GCCTACTTTCACCGGTAGAACCGAATGGCAATATGAGA (sense)  *HRE*-deletion Primer set  *CmsHSP21ΔHRE1*  Primers 29 & 35 as well as 30 & 36  *CmsHSP21ΔHRE2* Primers 29 & 37 as well as 30 & 38  *CmsHSP21ΔHRE1&2* Primers 29 & 37 as well as 30 & 38 |

^*^ The direction of primer sequence is 5′ to 3′.

^**^R = A,G; Y = C,T; M = A,C; W = A,T; H = A,C,T; S = C,G; V = A,C,G; B = C,G,T; N = A,C,G,T.
